# Quality and Performance Measurement in Primary Diabetes Care: A Qualitative Study in Urban China

**DOI:** 10.34172/ijhpm.2022.6372

**Published:** 2022-06-07

**Authors:** Alon Rasooly, Yancen Pan, Zhenqing Tang, He Jiangjiang, Moriah E. Ellen, Orly Manor, Shanlian Hu, Nadav Davidovitch

**Affiliations:** ^1^School of Public Health, Ben-Gurion University of the Negev, Beer Sheva, Israel.; ^2^Department of Epidemiology, Fielding School of Public Health, University of California, Los Angeles, CA, USA.; ^3^Shanghai Health Development Research Center, Shanghai, China.; ^4^Braun School of Public Health and Community Medicine, Hebrew University, Jerusalem, Israel. 5 School of Public Health, Fudan University, Shanghai, China.; ^5^School of Public Health, Fudan University, Shanghai, China.

**Keywords:** Quality Indicators, Primary Health Care, Diabetes Mellitus, China, CFIR, Implementation Science

## Abstract

**Background:** Quality measurements in primary healthcare (PHC) have become an essential component for improving diabetes outcomes in many high-income countries. However, little is known about their implementation within the Chinese health-system context and how they are perceived by patients, physicians, and policy-makers. We examined stakeholders’ perceptions of quality and performance measurements for primary diabetes care in Shanghai, China, and analyzed facilitators and barriers to implementation.

**Methods:** In-depth interviews with 26 key stakeholders were conducted from 2018 to 2019. Participants were sampled from two hospitals, four community healthcare centers (CHCs), and four institutes involved in regulating CHCs. The Consolidated Framework for Implementation Research (CFIR) guided data analysis.

**Results:** Existing quality measurements were uniformly implemented via a top-down process, with daily monitoring of family doctors’ work and pay-for-performance incentives. Barriers included excluding frontline clinicians from indicator planning, a lack of transparent reporting, and a rigid organizational culture with limited bottom-up feedback. Findings under the CFIR construct "organizational incentives" suggested that current pay-for-performance incentives function as a "double-edged sword," increasing family doctors’ motivation to excel while creating pressures to "game the system" among some physicians. When considering the CFIR construct "reflecting and evaluating," policy-makers perceived the online evaluation application – which provides daily reports on family doctors’ work – to be an essential tool for improving quality; however, this information was not visible to patients. Findings included under the "network and communication" construct showed that specialists support the work of family doctors by providing training and patient consultations in CHCs.

**Conclusion:** The quality of healthcare could be considerably enhanced by involving patients and physicians in decisions on quality measurement. Strengthening hospital–community partnerships can improve the quality of primary care in hospital-centric systems. The case of Shanghai provides compelling policy lessons for other health systems faced with the challenge of improving PHC.

## Background

**Key Messages**

** Implications for policy makers**

- In the studied setting, we found a rigid organizational culture between levels of the administrative hierarchy, which co-exists with an atmosphere of collaborative teamwork within the nested primary care institutes.
- Bottom-up and middle-out feedback can improve the quality of primary diabetes care and reduce the incidence of gaming the system among providers.
- Training of family doctors by hospital specialists and integrating community and hospital services are useful steps for improving the quality of primary care.
- We recommend that policy-makers include disparities impact assessments in quality evaluation reports, in addition to the current use of general performance thresholds.

** Implications for the public**

Quality indicators provide primary healthcare workers with benchmarks for improving diabetes care; however, little is known about how these are implemented in countries with hospital-centric health systems, such as China. In the studied setting, indicators were set in a top-down fashion by healthcare administrators, who consult infrequently with frontline primary care providers and patients on the design of indicators. Enhancing public involvement in the planning and evaluation of quality indicators is essential for tailoring quality measurements to patients’ needs and expectations. This can be attained by transparently reporting quality assessment results to the public, soliciting public feedback on program design, and including patient representatives in the decision-making process.

Primary healthcare (PHC) is a highly effective and people-centered approach for addressing the main causes of poor health and improving the wellbeing of individuals within their communities.^1^ However, in countries such as China, with a long history of hospital-centrality, patients often bypass primary care and seek tertiary care directly.^2,3^ While quality improvement is crucial for achieving the desired paradigm shift from tertiary toward primary care, a systematic review has shown that the practice of auditing and benchmarking against evidence-based guidelines is uncommon in low- and middle-income countries.^4^ Also, few studies have examined the contextual factors that determine how quality measures are implemented in these settings. Using diabetes as an example, in this study we describe the current state of quality measurements in primary care and analyze facilitators and barriers to their implementation in Shanghai, China.

 There are more than 537 million adults living with diabetes worldwide, with about one fourth of them in China.^5^ Compared with a reported prevalence of about 1% among China’s adult population during the 1980s, the prevalence rate increased to 5.5% by 2001, and further increased to 11.2% by 2017.^6-8^ Under-diagnosis is a major problem in China, with about 52% of individuals with diabetes being unaware that they have the condition, according to the 2021 International Diabetes Federation Atlas.^5^

 In addition to the condition’s increasing burden on individual wellbeing, studies show that the economic burden due to diabetes in China is substantial.^9,10^ According to one study conducted during 2015 in 16 tertiary hospitals in urban China, the mean annual total direct medical cost per patient with diabetes was US$1990, and the average cost per inpatient admission was US$2127.^9^ Furthermore, a nationally representative study of the quality of PHC for diabetes in China showed that diabetes-related hospitalizations have increased from about 4% in 2011 to 6% in 2013, while recurrent hospitalizations among individuals with diabetes increased from almost 19% in 2011 to 28% in 2015.^11^ As hospitalizations serve as a proxy outcome indicator for the quality of primary diabetes care, these findings highlight the urgent need to improve the level of care delivered by primary care practitioners in China.

 A strong PHC system serves as a prerequisite for diabetes care of excellent quality, according to the experiences of high-income countries where indicators are implemented.^12-15^ Although starting from a disadvantaged point, in recent years China has made significant progress in strengthening its PHC capacity. First, a remarkable increase in health insurance coverage has been achieved, from 29.7% in 2003 to 95.7% in 2011.^16^ By raising public funding, China has increased the accessibility and equity of health services, including PHC.

 Furthermore, significant changes have been made in PHC financing and the composition of medical staff salaries. The implementation of the Zero Mark-up Policy in 2011 has banned healthcare providers from increasing their income through the sale of prescription drugs.^17^ The new policy led to a sharp reduction in PHC revenue, with community and township health centers undergoing a 40% decrease in drug-related incomes, which also drastically affected physicians’ salaries.^18^ To compensate for this loss in revenue, as well as to strengthen PHC in general, between 2009 and 2015 the government increased its subsidies to PHC institutions from US $2.8 billion to US $20.3 billion.^19^ A proportion of these subsidies was distributed to PHC providers in the form of performance-based bonuses, which on average accounted for about 30% to 40% of providers’ new total income.^20^ Lastly, based on pilot experiences of local governments, in 2016 the Chinese government began to nationally implement a policy of “Family Doctor Contracting Services,” which is a form of gatekeeping used to encourage citizens to register with a family doctor who would then serve as their main point of care.^21^ To incentivize family doctors to contract with patients in this way, an annual sum was provided to doctors for each contracted patient. These sums varied by region, with higher average annual contracted service fees per patient in eastern China of US $20 in 2019, in comparison with the central (US $5) and western (US $7) regions.^22^

 Despite the progress made since the 2009 healthcare reforms, recent studies have emphasized several challenges facing PHC services in China. Between 2010 and 2018, the proportion of outpatient services provided by PHC institutions in China decreased from 62% to 53%, with the remainder being provided by hospitals.^23^ This figure is surprising considering the substantial increase in PHC subsidies since the healthcare reforms.^19^ The limited capacity and skills of PHC professionals were found to be the most common reasons (32%) why patients bypassed these institutions in favor of hospitals, according to a survey that covered 17 provinces in China.^24^ Patients’ trust in the quality of primary versus hospital care was consistently found to be a barrier to accepting the family doctor model, according to several quantitative and qualitative studies.^21,25-27^ A lack of interoperable health information systems and the fragmentation of service delivery between primary, secondary, and tertiary care providers were identified to be additional barriers to measuring and improving the quality of primary care.^19,28^

 The above findings, as well as the paucity of evidence about improvements in quality, highlight the need for an in-depth analysis of how quality indicators are implemented in China’s PHC context. Building on the health system framework adopted by Yip et al in their analysis of 10 years of healthcare reform in China,^29^ the starting point of the present study is that quality and performance measurement are not independent elements that can be easily singled out from the system. Rather, they are the intermediate and final products of policy levers (ie, financial, organizational, and regulatory arrangements) and healthcare delivery systems (ie, governance, incentives, management characteristics, and more). Therefore, the implementation of quality and performance measurements is intimately related to a system’s arrangements and characteristics, and therefore they must be analyzed within their policy and healthcare delivery context.^30^

 Bearing in mind the intricate relationship between measurement and context, the goal of this study was to answer two key questions: (*a*) what is the current state of quality and performance measurement in primary diabetes care, and (*b*) what are the facilitators and barriers to their implementation. To gain an in-depth understanding of these issues, we focused our investigation on Shanghai, where the local government was one of the first to implement the family doctor contract policy and to evaluate the quality of diabetes care provided.^21,31,32^

###  Study Setting and Policy Context

 PHC services in Shanghai are provided by a team of family doctors, nurses, and other health professionals, who work in 240 community healthcare centers (CHCs) and their affiliated community healthcare stations.^33^ The municipal health commission evaluates the performance of all CHCs, which are nested within the city’s 16 districts. Performance payments are allocated to CHCs based on indicators that measure structural and procedural aspects of CHCs, including, but not limited to, the availability of equipment, medical staffing, residents’ satisfaction with services and their signing rate with family doctors, vaccination rates, and the management of chronic conditions.^33^ In turn, CHC managers allocate performance payments to family doctors, nurses, and public health practitioners as bonuses, according to their performance. This scoring system is also aligned with national requirements and serves as a criterion for promotion for CHC- and district-level leaders.^34^

 In line with the national policies mentioned above,^17-20^ the salaries of family doctors in Shanghai comprise a basic wage, performance-based payments on both monthly and annual bases, and an annual contracted service payment. According to a survey conducted in Shanghai during 2015,^35^ half of the 153 family doctors who responded earned between 11% and 50% of their annual salary as performance-based payments, and about a third of the responders earned more than 50% of their annual salary as pay-for-performance. These figures varied among family doctors according to their seniority and district.^35^ For example, a senior family doctor in an urban district who was interviewed for this study estimated the proportion of monthly and annual performance-based payments to account for about 40% and 30% of his total annual salary, respectively, with the basic wage and annual contracting fee comprising about 20% and 10%, respectively.

 Of note, several features of Shanghai’s pay-for-performance scheme in PHC coincide with the UK’s Quality and Outcome Framework (QOF). These common features include the use of indicators, incentives, and weighted scoring systems (both with a sum of 1000 points) to augment family doctors’ performance. This resemblance may be due to numerous Chinese articles about the QOF that were published and which included recommendations for policymakers in China to implement incentive schemes for primary care that are similar to those of the United Kingdom,^36-39^ and specifically in Shanghai.^38,39^

 The three indicators used by the Shanghai Health Commission to measure primary diabetes care originated from China’s national basic public health service standards, the first edition of which was published in 2009, and its subsequent editions.^40,41^ The first indicator, *health management rate*, is calculated as the number of residents with diabetes who are treated by a CHC’s health workers divided by an estimate of the number of people living with diabetes within the CHC’s catchment area. The second indicator, *standardized management rate,* is the number of individuals with diabetes who receive quarterly health follow-ups by the CHC medical team as a proportion of residents with diabetes who receive care at the CHC. The third indicator, *glycemic control rate,* is the number of individuals with diabetes with fasting blood glucose <7 mmol/L at their latest follow-up divided by the same denominator as for the diabetes standardized management procedure. According to the 2019 evaluation of community healthcare services in Shanghai,^42^ the standardized management rate was 87.3% (required standard threshold >60%), and the glycemic control rate was 64.54% (required standard threshold >40%). It should be noted that the *health management rate* indicator was removed from Shanghai’s pay-for-performance scheme and replaced by the *glycemic control rate *indicator in 2016.

## Methods

 In line with the above aims, we used an interpretivist research paradigm and a qualitative thematic approach for data collection and analysis.^43,44^ The interpretivist perspective was considered suitable because quality measurement is dependent on how decision-makers define quality and how it is interpreted by other stakeholders (ie, physicians, administrators, and patients) in a unique context. Furthermore, the Consolidated Framework for Implementation Research (CFIR) was combined with the above to guide data collection and analysis. CFIR is a meta-theoretical framework that provides a comprehensive listing of constructs organized across five major domains^45^: intervention characteristics, inner setting, outer setting, characteristics of individuals, and process. The widespread use of the CFIR typology in implementation research, and in the implementation of quality measurements in particular, provides an advantage when making comparisons across the contexts of various health systems.^21,46-48^

 The intervention in this study was defined as the tools for quality and performance measurement implemented in PHC in Shanghai, along with the supportive policies that were considered to be prerequisites for such measurements in PHC. For example, policies that encourage and support the use of PHC services, such as the family doctor contract policy, are considered part of the intervention. This is because, in the hospital-centric context of this study, patients tend to bypass PHC services in favor of directly seeking care in hospitals,^25,49^ and without such policies quality measurement in PHC would be far less meaningful. In terms of other CFIR domains (ie, “inner setting,” “outer setting” etc) our analysis was purposefully broad, to include policies and system components that were related to the measurement policies, from the perspective of the stakeholders interviewed.

 Lastly, the Standards for Reporting Qualitative Research (SRQR) were used as guidelines for presenting this study’s findings.^50^ SRQR consists of 21 items, which were used to assess and revise the manuscript’s key sections, from the abstract to the discussion. The rationale for using SRQR derives from the qualitative nature of this study and because the use of these standards can facilitate judgments about the trustworthiness, relevance, and transferability of the findings from this study to other contexts.

###  Sampling of Key Informants

 We conducted in-depth interviews with stakeholders involved in providing, regulating, or receiving diabetes primary care in Shanghai. In terms of sampling strategy, we conducted in-depth interviews at two tertiary hospitals and four CHCs; at least one physician and two patients were interviewed at each institution. As healthcare in China is provided by both traditional Chinese medicine (TCM) and Western medicine (WM) practitioners,^51^ and some hospitals provide TCM in addition to WM, we selected one TCM–WM-integrated hospital and one WM-only general hospital. The four CHCs were randomly selected from eight CHCs in the district where the study was conducted. In addition, we contacted policy-makers involved in regulating the quality of primary diabetes care in Shanghai, from the following administrative levels: district health bureau, municipal health bureau, municipal center for disease control, and a research center for diabetes policy. The selection of policy-makers for the interviews was based on a convenience sample.

###  Data Collection and Analysis

 The interview guide included open-ended questions, tailored according to the stakeholder group interviewed. For physicians, CHC managers, and policy-makers, open-ended questions were grouped according to the five domains of CFIR, to allow greater emphasis on policy and inter-stakeholder interactions. The interview guide for patients focused on their lived experience within the health system and consisted of open-ended questions within three domains: living with diabetes, receiving care at a CHC versus a hospital, and questions on policy. The interview guides are available in Supplementary files 1 and 2.

 The in-depth interviews were conducted during 2018 and 2019; they were jointly conducted by two of the authors, one of whom is from China. Working as a bi-national team enabled accurate and context-specific data collection in Chinese, while also facilitating an external audit and critical interpretation of the implementation context. After obtaining a participant’s informed consent, the interview session was conducted in Chinese, audio-recorded, then transcribed, first by an automated, online speech-to-text service, and then verified line-by-line by one of the authors. This process assured the quality of translation, in alignment with the original audio recordings. Timestamps were added to the text prior to translation into English, to facilitate quality control of the Chinese to English translation process.

 Thematic analysis was conducted in parallel, in English and Chinese, using ATLAS.TI version 8. Each author independently coded the interview transcripts, using CFIR constructs as codes when deemed relevant to the text, while simultaneously using open coding when interview excerpts did not directly match one of the CFIR constructs. Toward the end of the coding process, the two authors discussed and compared codes with each other and made modifications as appropriate, such as combining similar codes. The ATLAS.TI’s “Network” function was used to make connections between CFIR codes and other codes grounded in the text, to develop the study’s key themes (see Figure S1, Supplementary file 3).

 To ensure analytic rigor and validity, the authors discussed the themes, and the findings were triangulated.^52,53^ This was achieved by matching interview excerpts voiced by multiple participants from different stakeholder groups (ie, family doctors, patients, and policy-makers) of relevance to the same theme (eg, PHC is cost-effective but under-financed).

 Lastly, the authors conducted member checking sessions with participants from each stakeholder group; these participants commented and gave feedback on the interpretation of the qualitative findings.^53^

## Results

 Our study included in-depth interviews with 26 stakeholders involved in providing, regulating, or receiving primary care for diabetes in Shanghai. Stakeholders included patients with diabetes (abbreviated as “P,” n = 12), family doctors (“FD,” n = 3), endocrinologists (“E,” n = 2), CHC managers (“M,” n = 4), and policy-makers (“PM,” n = 5). Details about the study’s participants are included in Tables S1 and S2 (Supplementary file 3).

 To assist in drawing comparisons between the context we studied and other health systems, our findings were organized and presented using constructs from the CFIR. Table includes brief descriptions of these constructs within the studied context, along with supporting quotes from the stakeholders interviewed. Constructs were presented under the five CFIR domains, in accordance with the original categorization proposed by Damschroder et al.^45^ Next, these constructs were consolidated in a thematic matter and are presented in greater depth later in the results section. Lastly, key barriers and facilitators for the implementation of quality measurement in primary diabetes care in Shanghai were summarized (see Table S3, Supplementary file 3) and further addressed in the discussion.

**Table:** Stakeholder’s Quotes on Primary Diabetes Care and its Measurement in Shanghai, Categorized According to CFIR Constructs

**CFIR Construct**	**Brief Description**	**Supporting Quotes**
**CFIR Domain: Process**		
Planning	Central government plans indicators via a top-down hierarchical process. Insufficient bottom-up feedback from frontline clinicians and middle- managers when planning indicators.	*“Our indicators are planned in accordance with national requirements… the municipal level will be blamed if the requirements are not met, just like failing an exam” *[PM05]. *“Decisions are made by the policy-makers... they will inform the medical staff on the standards… in fact, we [medical staff] are all involved in the completion of quality indicators, but do not participate in their development” *[M04].
Reflecting and evaluating	The municipal health commission monitors CHCs’ performance via an online application (App). Frequency of evaluations has increased from yearly to daily monitoring of family doctors’ work.	*“The App is a comprehensive information platform for the Shanghai community health service reform… Over 200 CHCs in Shanghai are ranked... our stress is relatively large. But we are doing quite well in management of chronic disease”* [M03]. *“The directors of the health planning commission in each district will take a look at the system [App] every day… they can see every family doctor on this app and how much work has been done today”* [PM05].
**CFIR Domain: Inner Setting**		
Goals	Goals are converted to pragmatic tasks via the quality indicators. Indicators cause stress, but result in greater achievements.	*The quality control system helps to continuously improve diabetes management and achieve better standards” *[E01]. *“[The indicator system] tells us clearly what our goals are and what we should do ... It exerts pressure on us, but it’s quite good since it lets us do more purposeful work”* [M03].
Culture (organizational)	Health workers in CHCs have a strong feeling of unity and there is a collaborative atmosphere. CHCs resemble cohesive troops within a “militarized” structure.	*“The [CHC] work atmosphere is very good. We eat lunch together every day, and at this time we will chat and discuss patients… we are really like a big family, our team leader cares about us, and we are quite harmonious” *[FD03]. *“Administrative management is relatively rigid. We seem to be a bit militarized. Sometimes there is no room for discussion”* [PM01].
Network and communication	Specialists support the work of family doctors by providing training and patient consultations in CHCs. A paradigm of hospital superiority is observed in specialists’ and family doctors’ relations.	*“Doctors in higher-level hospitals provide consultations in our CHC every two weeks, and a family doctor joins the specialist…Specialists also come to our community every month to train family doctors” *[FD01]. *“Because tertiary hospitals do not have enough manpower, the future trend is to ‘sink’ patients with diabetes into CHCs for management, and a lot of work is done by the community. So two senior doctors from tertiary hospitals go to CHCs to do training” *[E02].
**CFIR Domain: Outer Setting**		
Peer pressure	Indicators are used to rank districts within the municipality and CHCs within the district. Managers differed in their attitude for ranking and motivation to excel.	*“In the past few years, our center did a good job for managing diabetes. Among the district’s eight CHCs our ranking was quite high. This propels us to work for higher achievements” *[M01]. *“The competition is meaningless. We are required to complete the work, if the work is done well, it will be fine; if it is not done well, we will be criticized” *[M02].
External policy and incentives	Family doctors are financially rewarded when excelling on measured indicators. Pressures to achieve targets may lead to data manipulation. Current policies permit patients to bypass CHCs and self-refer to hospitals.	*“The quantity and quality of diabetes visits are standardized, and in the end, all our work is combined. For me, the performance incentive is relatively high”* [FD01]. *“There are some doctors, a small number of doctors, who sometimes may use certain ways to manipulate a patient’s indicator into the normal range”* [M04]. *“In Shanghai there is no mandatory gatekeeping, so patients can go directly to the higher-level hospital…it is actually because of not trusting family doctors” *[PM05].
Patients’ needs	Distrust in family doctors and their clinical capabilities leads patients to tertiary hospitals, circumventing CHCs. Gradual establishment of trust, ongoing policy transitions, and hospital waiting times encourage patients to seek care from their family doctor.	*“I always doubt CHC doctors’ ability and their accuracy of disease judgment… I am afraid of misdiagnosis, so I first consult the doctor in the tertiary hospital... But in terms of service attitude, family doctors in CHC are better. We are acquaintances and familiar with each other” *[P08]. *“I don’t need to go to a tertiary hospital now. Under the management and guidance of my family doctor, my condition is very stable… I have paid more attention to my diet and living habits, so there is no need to refer. Because there are so many people in big hospitals, even if the family doctor advises me to go, I am not very willing” *[P05].
**CFIR Domain: Individual**		
Knowledge about intervention	Awareness regarding the advantages of CHCs is perceived as necessary for seeking care there, a condition for quality measurement in CHCs.	*“Because of the nature of my work, I would be willing to go to a family doctor. But my classmates, if they don't know about CHC and primary care, they definitely prefer tertiary hospitals, and even skip secondary hospitals. They have money and need the best therapy. The key is they don’t believe family doctors’ ability to diagnose and treat” *[PM02].
Support of intervention	Family doctors support the quality evaluation system, perceiving it to be a “scientific” and effective way to provide diabetes care.	*“Relying only on the communication between us and patients without strict data evaluation, I think it’s unscientific… through rigorous big data research, it is more effective and meaningful for us to complete the control of diabetes. Such a quality evaluation system is currently most effective for patients with diabetes” *[FD02].
**CFIR Domain: Intervention Characteristics**		
Cost	Insurers and patients benefit from adequate primary diabetes care. Under-financing of accurate glycemic tests (HbA1c) restricts family doctors’ ability to improve care.	*“From the data of medical insurance in the past two years… family doctors have indeed played a role in controlling fees through the management of chronic diseases” *[PM02]. *“I think that the control rate for diabetes is still not enough. HbA1c is the standard [test]. If patients could have tested it for free, it is good but not realistic” *[FD03].
Trialability	The hierarchical structure supports piloting policies and technologies in a subset of CHCs before wider implementation.	*“When we started doing this project, I didn’t know what kind of problems we will encounter during the execution… I chose one or two CHCs to do this with us... If they work with us and make good results… we will convert it to standard into a policy, and then all CHCs would do it”* [PM04].

Abbreviations: PM, policy-maker; M, CHC manager; E, endocrinologist; FD, family doctor; P, patient; CHC, community healthcare center; CFIR, Consolidated Framework for Implementation Research; HbA1c, hemoglobin A1c.

###  Top-Down Goal Setting and Indicator Planning

 According to our interviews, health administration in China is highly hierarchical, with goals and indicators being set at a national level. These are then contextualized to the provincial or municipal level and passed on to the district level, where staff operationalize the goals to specific requirements set for health workers in CHCs. As can be seen from the quotes in Table under the “planning” construct, indicators and policy decisions are formed with little involvement of the frontline health workers who they affect the most. This perspective was prevalent among most of the CHC managers and family doctors interviewed, although the interviewees varied in their degree of accepting this reality of exclusion.

 Additionally, the degree to which family doctors attain the goals prescribed by policy-makers is continuously monitored through the use of an online app (see “reflecting and evaluating” in Table). Top-down dynamics were deemed to be tacit knowledge among the study’s participants or, in other words, part of the all-encompassing organizational culture in which community healthcare services operate.

 Policy-makers who also serve as middle managers in municipal- and district-level health organizations were aware of the unidirectional process of decision making. This has led them, in some cases, to seek the opinions of health workers and patients at the community level. However, it was noted that such practices are not part of the formal decision-making process, which was described as “*rigid*” and “*militarized*” by one stakeholder (PM01).

 “*The entire process only goes top-down, without information going up from the bottom levels. From a management point of view, effective management must connect upstream and downstream information … What are the problems encountered by people working at the community level? Whether the indicator’s data is really collected, and whether the means of collecting data is clear for people in the upper level*”[PM05].

 According to interviewee PM05, a lack of “*upstream*” information from the community level can also lead to ineffective management. There appeared to be some ambiguity as to what extent the indicators collected reflected the exact situation on the ground, an uncertainty which could have been addressed through better bilateral and mutual communications. It is worth noting that while the organizational culture was described as rigid, militarized, and lacking bottom-up input, there was still room for piloting new policies and interventions within the hierarchical management framework (see the quote from interviewee PM04, under “trialability” in Table).

###  Divergent Peer-Pressure Effects 

 Among our interviewees, managers differed in their attitude toward the peer pressure exerted through indicator-based ranking. For some (eg, M01, quoted in Table), ranking amplified their motivation to excel at the measured indicators. For other managers, as long as the indicator threshold values were attained there was little pressure or interest to pursue higher standards. This attitude can be noted in the quote from interviewee M02 (Table), as well as that of interviewee M04:

 “*There will be ranking within the district, like the districts are ranked among themselves … overall, the pressure is not so great, because the work we do is the same, and the standards that need to be fulfilled are the same. I personally feel that the pressure is not great. There is no competitive relationship*”[M04].

 These views by interviewees M02 and M04 contrast with those expressed by interviewee M01 (Table), who stated that they have performed well in diabetes indicators in the last several years and were motivated to maintain high standards. Such discrepancy in attitudes was explained by one of the policy-makers interviewed:

 “*Some districts don’t want to work very hard to get the money…there is actually a stratification phenomenon. For districts that always do a good job, the first five districts want to be ‘Number 1’ to receive rewards from their leaders. They will be willing to work very hard because they have a chance to stand out. For the lowest (ranked) districts, such as numbers 14, 15, 16, they are willing to work hard because they don’t want to be last. However, for those in the middle, it’s different. They think that they can neither go up, nor fall down, so they don’t have much motivation. In China, your position determines your mindset*” [PM05].

 The above findings suggest varying effects of peer pressure on improving performance in Shanghai’s PHC system. High- and low-performers may be more motivated to excel than mid-range performers in the setting we studied.

###  Pay-for-Performance – a Double-Edged Sword

 PHC providers are financially rewarded when excelling on measured indicators, while they are also subject to penalties when under-performing. These incentives are orchestrated in unison with the evaluation system, set in accordance with the national goals presented earlier. The performance incentives create a “*source of urgency and motivation*”(FD01) for family doctors as they strive to increase their income as well as their center’s relative rank in comparison with other CHCs in the municipality.

 Beyond their intended consequences, financial incentives were also associated with false reporting. As can be seen from the quotes in Table for the “external policy and incentives” construct, by using indicators the health commission pressures family doctors to achieve certain targets. In some cases, when the target threshold is deemed beyond reach, unintended consequences may occur, such as manipulation of a patient’s indicator to the desired range. Another type of unintended consequence was the inclusion of healthy patients as having diabetes. This practice was attributed to the “health management” indicator, which was removed from Shanghai’s pay-for-performance scheme in 2016. However, it still serves as a case study for the complex relationship between policy-makers’ intentions, unintended practices by physicians, and subsequent policy readjustment.

 “*In the first few years, funds were allocated according to the ‘health management rate’, which means CHCs received payment according to the number of people they manage… What would happen in this situation? In order to get the money, many people reported false figures… Many people with diabetes appeared, but in fact these people did not suffer from diabetes. They [doctors] did this to get the funds. And now the country has changed to [measuring] standardized management…*”[PM05].

 As the health management indicator relied on the number of patients receiving management, it was easily manipulated by including “*people who did not suffer from diabetes*”(interviewee PM05) in CHC management cohorts. Once the issue of false reporting had been identified, policy-makers shifted from measuring health management to measuring standardized health management, as the latter depends on providing quarterly care to residents with diabetes who are already managed by the CHC. Using the standardized management indicator, therefore, was seen as a solution to the unintended practice described above.

 Additionally, several policy-makers who were interviewed were aware of the phenomenon of false or incorrect reporting in primary care and noted that quality control mechanisms had been introduced. These mechanisms include data verification, telephone interviews with patients, and random inspections and on-site visits, as noted in the quotes below.

 “*Suppose this person [doctor] tells me he measured HbA1c on a certain day, but when I checked the ‘diagnosis and treatment platform’ I could not find the result in the system. Then, I will doubt the accuracy of the result, and will further check the CHC information by randomly selecting some patients. If I think this part of the data is abnormal, I will follow up and call to verify: ‘Did the doctor give you the service that day?’... If the CHC told me that this person participated in chronic disease management service that day, I will check their registration system to see if the patient really came. Then I will report the results to the health administrative department of their district*” [PM04].

 “*We have random inspections, including for service quality, and we sometimes check cases directly… So how do I check a doctor? After a patient has been diagnosed, we ask for his health card to check what the doctor recorded … If the patient said that a certain process did not occur, then the [doctor’s] score will be deducted… this is to ensure that the entire process is true and effective*”[M01].

 According to the quotes above, from interviewees PM04 and M01, policy-makers and managers were engaged in numerous quality control activities in order “*to ensure that the entire process is true and effective*” (interviewee M01). Instances in which there were discrepancies between what the doctor reported and the information verified with patients were handled by reporting the incidents to the district-level health authorities (interviewee PM04) or by directly reducing the physician’s score (interviewee M01), which determines performance-based salary. While changing the incentivized indicators and increasing scrutiny was perceived as being useful for reducing false reporting, the quote below suggests that physicians’ methods have become more sophisticated.

 “*… And now the country has changed to [measuring] standardized management … then the ‘virtual number’ was reduced a lot … in the past, he [the doctor] only needed to report a number. Now even if this person does not exist, he still has to report four follow-up records every year. This has increased the cost of his fraud … Then the country continues to ‘squeeze out the moisture from the data’ by making another indicator called blood sugar control rate … in this way they can see whether your [the patient’s] blood sugar level is real and effective … However, there will still be false situations where, in fact, fraud is quite simple. Change the name, or merge the first half of this person’s case with the second half of that person’s medical record*”[PM05].

 The interview excerpts above suggest that the enacted policies and top-down supervision have “*increased the cost*” of false reporting but have not eliminated this possibility altogether. Interestingly, the quote from interviewee PM05 suggests that policy-makers are seeking new ways to increase data validity (“*squeeze out the moisture from the data*”) and that the revision and addition of new diabetes indicators are informed by such considerations.

###  Patients’ Experience of Community and Hospital Care

 According to our findings, in recent years CHCs have improved at addressing certain aspects of patients’ needs, particularly for patients who signed up with family doctors. For example, drugs that were previously only available at tertiary hospitals were made available in CHCs for longer prescription periods and at a higher reimbursement rate. This change in policy supported family doctors’ efforts in reaching out to more patients, particularly those who require medication for a chronic condition. By doing so, the combined efforts of policy-makers and family doctors have created more opportunities for health education, counseling, and management of chronic conditions.

*Interviewer: *“*What is the difference after signing with a family doctor?*”

*Patient: *“*It is more convenient when prescribing medicine. Sometimes, the family doctor calls and invites us to join lectures on diabetes. There is also a free physical examination once a year, which includes ultrasound and blood glucose measurements. The CHC organizes, and the neighborhood committee informs us that people over the age of 60 can go to the neighborhood committee to test the blood glucose indicator*”[P06].

 Despite the progress made, distrust in family doctors and their clinical capabilities remains a persistent barrier to seeking care in CHCs rather than tertiary hospitals. Even among patients who were interviewed in the community, we witnessed reluctance to fully depend on family doctors (Table, quote from interviewee P08). However, other patients we interviewed suggested that trust is gradually being established (Table, quote from interviewee P05). According to findings presented under the “networks and communications” construct (Table), specialists support the work of family doctors by providing training and patient consultations in CHCs. Patients were supportive of this policy as it allowed them to see specialists in the community setting.

 Family doctors were considered to be better in terms of their service attitude and their ability to assist patients in changing their life habits. In doing so, they were deemed more relevant for assisting patients with chronic diseases to manage their medical conditions. While endocrinologists in tertiary hospitals were perceived as having clinical superiority, their consultations were considerably shorter and waiting times were longer. Therefore, as patients became more familiar with their family doctors’ role, more of them preferred to receive care in CHCs.

 Quality indicators were perceived by some interviewees as disconnected from how patients experience their own health. This meant that while policy-makers and managers who were interviewed generally agreed on the advantages that indicators have for improving patients’ clinical health, some recognized that a certain distance exists between what they perceive to be a good quality of care and what may be considered so by patients. This can be seen as an issue of intervention design, as the indicators did not fully capture the quality of care as experienced by patients.

 “*We pay less attention to health as experienced by the patient. It is very simple now, when measuring satisfaction, the patient fills a paper, and checks some items… Did he actually improve in his psychosocial state? Or is he anxious all day because he gets sick of going around for treatment? We hope to actually see such a concept… an improvement in the experience of the entire patient*”[PM05].

 According to the findings from this section, the interviewed stakeholders suggested that there is an ongoing transition of the health system toward better accommodating patients’ needs. However, important gaps remain. These include family doctors’ clinical capacity, patients’ trust in the capabilities of CHCs, and the need for the formulation of quality indicators that better capture patients’ perspectives of their own health status.

###  Primary Healthcare Measurement Is Cost-Effective but Under-financed

 The role played by family doctors in reducing costs – for both patients and the insurance bureau – was acknowledged by policy-makers who participated in our study (Table, quote from interviewee PM02). Stakeholders highlighted the importance of increasing the quality of PHC services, as most diabetes-associated complications can be prevented or reduced with adequate glycemic control.

 An important cost-related barrier to improving the quality of primary diabetes is public financing of hemoglobin A1c (HbA1c) tests, which are used to accurately assess glycemic control. Currently, patients’ glycemic control in China is mostly assessed by measuring their blood glucose levels. However, this method is considered inaccurate, as blood glucose levels fluctuate throughout the day. The HbA1c test, on the other hand, provides a measure for assessing a patient’s average glycemic control during the past three months. At the time the study interviews were being conducted, the reasons why the HbA1c test was less commonly used in CHCs were the costs of implementation and the costs of testing incurred by patients, which are partially reimbursed.

 “*We require everyone to do it [HbA1c] in principle every year. However, HbA1c testing is more expensive. It costs about 60 yuan for a test, while doing one for blood sugar is 5 yuan, so not everyone is willing to do HbA1c*”[PM04].

 “*So now that I am discharged from the hospital, I plan to come here every year to do a comprehensive examination aiming to the symptoms of diabetes. Because CHC can’t do some exams, such as HbA1c. CHC can only prescribe common drugs and conduct routine tests*”[P12].

 To summarize, diabetes-associated hospitalizations are a worrisome outcome that can incur high costs for the individual patient as well as insurers. Even though many episodes of hospitalization could be prevented by adequate glycemic control in the community setting, the under-financing of accurate tests limits family doctors’ ability to improve their quality of care.

###  Key Barriers and Facilitators

 The main facilitators and barriers to the implementation of quality measurement in primary diabetes care in Shanghai are summarized in Table S1. Notably, indicators were uniformly implemented via a top-down process, with daily monitoring of family doctors’ work and pay-for-performance incentives for attaining national and local goals. While the patients interviewed had an initial preference to directly seek tertiary rather than primary care, we found that CHCs have improved at addressing patients’ needs and that some patients are gradually establishing trusting and continuous relationships with their family doctors.

 Key barriers found in our study include the exclusion of frontline clinicians from indicator planning, a lack of transparent reporting on the quality of care patients receive, and a rigid organizational culture that leaves little room for bottom-up feedback. The issue of false reporting arose in some cases, particularly where the required thresholds were perceived to be beyond the reach of family doctors. Additionally, current indicators are not adjusted to patients’ social-demographic characteristics. Stakeholders also noted that further government investment is required to improve the clinical appropriateness of indicators and increase the level of quality measurements to international standards.

## Discussion

 After more than a decade of health reforms, China has made considerable progress in the dimensions of social health insurance, structural investments in PHC facilities, and implementing a family doctor healthcare delivery model.^19,21,29^ However, little research has been published into the government’s approach to quality improvement in primary care, as perceived by different stakeholders. By analyzing the case of primary diabetes care in Shanghai, whose local government pioneered China’s recent PHC policies, we provide novel insights into how quality indicators are implemented in the Chinese PHC context.

 In the studied setting, we found a rigid organizational culture, with quality measurements and associated policies being developed by China’s central government and passed down the hierarchical ladder. However, even within this rigid structure, there were several points of flexibility. Innovative and novel policies were being piloted in some CHCs, giving district- and municipal-level policy-makers an opportunity to consider wider implementation strategies through a process of trial and error. While pay-for-performance incentives exerted pressure on CHCs to compete, we found that within each CHC there was an atmosphere of collaborative teamwork. Lastly, we noticed a complex relationship of competition between CHCs and hospitals, as these entities gradually shift from competing for patients to collaboratively improving the quality of primary care.

 Our investigation shares several findings with other studies conducted in China where CFIR was applied to analyze barriers and facilitators to implementation. In their study on the implementation of evidence-based public health in China, Shi et al^54^ found that “although external policies proposed that practitioners implement evidence-based practices… government funding was insufficient.” This finding coincides with the “cost” barrier in our study for implementing HbA1c testing in the community, although other evidence shows that HbA1c levels are the strongest predictor of myocardial infarctions and stroke among patients with diabetes.^55^ It is important to note that major improvements in access and standardization of HbA1c testing have been achieved in China. For instance, the number of laboratories that participated in the Shanghai-led “Glycohemoglobin Harmonization Program” increased from just 9 laboratories in Shanghai itself to 192 laboratories throughout China between 2010 and 2018.^56^

 Access to advanced testing and expertise in primary care can be further improved by strengthening partnerships between CHCs and tertiary hospitals, as the latter can share medical resources and professional knowledge with health practitioners who serve the community. Findings included under the “network and communication” CFIR construct showed that specialists support the work of family doctors by providing training and patient consultations in CHCs. According to other studies conducted in Shanghai, similar approaches have shown great success both within an integrated hospital–community diabetes management program and in a community-based colorectal cancer screening program.^32,57^ In many low- and middle-income countries, tertiary facilities have more resources at their disposal, while primary care institutes have better access to the community; therefore, strengthening hospital–community partnerships can have a profound impact.^2^

 Within CHCs, our findings suggest that CHC managers prioritize an organizational culture of team effort and collaboration among community health workers. For managers of CHCs, assuring “*team cohesion*”(interviewee M04)was appreciated as a high priority, while for family doctors, leadership that encourages cooperation and concern toward PHC staff produces a “*harmonious*” atmosphere (interviewee FD01)and feelings of being part of a “*big family*”(interviewee FD03). These perceptions were noted among interviewees from all CHCs included in the study, thus our findings suggest that this collaborative culture may be widespread. While these findings may contrast with the “*rigid*” (interviewee PM01) administrative culture outside of CHCs, the analogy used by the district leader quoted may appear to be fitting, as CHCs can be interpreted as cohesive troops within the “*militarized*”(interviewee PM01)hierarchical structure.

 We compared and contrasted the results of our study with the results of a study conducted by Yuan et al,^21^ who used CFIR to analyze barriers and facilitators for implementing the family doctors’ contract policy in China. Similar to our study, they concluded that “based on the CFIR framework … in the inner setting, more attention should be paid to the quality of primary care and the competency of family doctors.” However, while in our study we found a rigid organizational culture of top-down decision making, Yuan et al noted that “the implementation of the family doctor contracting services in China involved a combination of both top-down and bottom-up processes.” One possible explanation for this discrepancy is that the “bottom level” defined by Yuan et al was local government, while patients were not included as participants in their study. Also, it is possible that family doctors were included in decisions regarding the contracting policy but excluded from the indicator planning discourse.

 According to a systematic review on implementation processes and pay-for-performance in healthcare, the engagement of stakeholders from all levels is critical to program development.^58^ Under the CFIR “outer setting” domain, the authors of the review noted that programs should have the capacity to change over time in response to provider input and maintain flexibility to meet the needs of their patient populations. In an OECD (Organisation for Economic Co-operation and Development) report on lessons learned about healthcare care quality from the experiences in 15 countries, the authors underlined that a strong patient voice should be prioritized^12^. In light of our findings, we suggest that patient and physician involvement in indicator planning and evaluation should be tailored to China’s context.

 Organizational culture in China differs from other countries in terms of the priority given to top-down decision making and its balance with bottom-up feedback. A compelling case to illustrate this issue is the introduction of massive public health interventions, including lockdowns, by China’s central government during the first wave of coronavirus disease 2019 (COVID-19) infections in January 2020.^59^ In response to the emerging pandemic, strategic decisions were made by the Central Leading Group for COVID-19 Prevention and Control, which was headed by China’s Vice-Premier. Directives made by the Central Leading Group were considered as non-negotiable political tasks to be fulfilled by all participants, via a fairly rigid and clear chain of command.^60^ That being said, the role of local agents was not merely to execute policies. Local policy activism and experimentation were encouraged, as they were able to generate innovative and contextualized solutions for policy problems.^60,61^ One example is the rapid conversion of stadiums to shelter hospitals in Wuhan during the pandemic, a solution that was later replicated in other cities and provinces.^62^ In the literature, this process is termed “experimentation under hierarchy,” which was defined by Heilmann as “a combination of decentralized experimentation with ad hoc central interference, resulting in the selective integration of local experiences into national policy-making.”^63^

 Findings from our study presented under the CFIR construct “trialability” suggest that experimentation under hierarchyalso applies to the context of PHC in Shanghai. Our findings further resonate with those of other studies, for example where district-level health administrators have assumed leadership positions in the introduction of a pilot program for colon screening in Shanghai and where CHC managers have played vital roles in the implementation of China’s family doctor contracting policy.^21,57^ Within the Chinese context, strengthening middle-outpathways in health policy can help to balance the dominant influence of top-down decision making and enhance the appropriateness of quality improvement initiatives to patients’ needs.^64^

 Utilizing the CFIR framework in this study allowed comparison with investigations into quality improvement from other countries where CFIR was utilized. Nouwens et al^47^ and Eldh et al^48^ used the framework to identify constructs associated with the implementation of an accreditation program in primary care in the Netherlands and to establish a national quality registry in Sweden, respectively. Although the studies differed in their settings, when considering the CFIR construct “reflecting and evaluating,” both arrived at similar conclusions: healthcare providers should receive timely, comprehensible, and accessible feedback on their quality of services. This is in agreement with the findings from our study, where stakeholders viewed the online evaluation app that provides daily reports on family doctors’ work as a facilitator to improve the quality of diabetes care in Shanghai.

 Findings under the construct “organizational incentives” suggested that current incentives function as a double-edged sword. On one hand, the pay-for-performance incentives were thought to increase family doctors’ motivation to excel on measured aspects of care. On the other hand, several policy-makers noted that incentives may increase family doctors’ propensity for fraudulent reporting to gain financial bonuses. Such practices were not unique to our setting. Studies conducted in the United Kingdom, the United States, and other countries have found that by “cherry-picking” the patients most likely to perform well on selected measures, physicians gamed the system without improving the care for some patients with diabetes.^65-68^

 Pay-for-performance incentives in Shanghai constituted about 11% to 50% of family doctors’ salary for about half of the survey respondents, while a third of respondents earned more than 50% of their annual salary as pay-for-performance in 2015.^35^ Considering this study’s findings, reducing the weighting of pay-for-performance incentives, while increasing family doctors’ baseline salary instead, could be an appropriate approach.^58^ Also, standardizing performance thresholds to individual’s characteristics, such as residents’ age and socio-economic status, can improve the clinical adequacy of indicators and further reduce the impetus on physicians to game the system.^65-67^

 As the evaluation system used in Shanghai was likely influenced by the UK’s QOF,^36-39^ policy-makers in China should also be aware of the framework’s limitations, as identified in the United Kingdom. For example, once the targets for incentive payments have been fully met, additional health gains from the QOF program become negligible.^69^ Also, the use of incentive target thresholds in the QOF was not associated with a significant reduction in mortality nor did they appear to address important disparities in diabetes management.^70-72^

 In several high-income countries, quality indicators in community healthcare are publicly reported and adjusted according to disparity indices such as socio-economical position.^12,14,15^ This allows providers, policy-makers, and the general public to understand not only the general trend in quality improvement but also whether and to what extent reducing health inequalities is successfully achieved. However, CHCs in Shanghai are currently evaluated solely on whether they attained the target threshold, and results are published only in internal reports.^33^ We therefore recommend that policy-makers include impact assessments of disparities in their performance analyses and make their reports more transparent to the public.^12,73^ By focusing on closing the quality chasm between residents with high and low socioeconomic status, policy-makers will be better equipped to address the social determinants that affect health and wellbeing among people with diabetes.

###  Study Limitations

 Our qualitative investigation was conducted among stakeholders in Shanghai who were mostly from the same district, therefore the generalizability of our findings may be somewhat limited. Rural or less affluent areas in China may experience other barriers to quality measurement in primary care that were not discussed in our study and will therefore require further research. Also, considering the relatively small sample used in our study, future quantitative or mixed-methods studies on the implementation of quality indicators for primary diabetes care are warranted, as these will enable our qualitative results to be statistically validated or refuted.

## Conclusion

 Measurements in primary care that can improve the quality, equality, and cost-effectiveness of medical services are strategic requirements for individual wellbeing as well as for the sustainability of national health systems. In this article, we performed a policy analysis to assess how the quality and performance of primary diabetes care is measured in Shanghai, from the perspectives of patients, doctors, managers, and decision makers. This study provides several lessons for policy-makers in China, as well as in other countries. First, the implementation of quality and performance indicators requires consideration of how other stakeholders might react to any new policies. Careful and early appraisal is particularly important when implementing pay-for-performance measurements, as these may function as a double-edged sword, leading to both intended and unintended consequences. Second, bottom-up feedback from frontline providers and middle-out leadership by regional managers can improve decision maker’s ability to initiate policy adjustments in a timely manner. Including PHC providers and patient representatives in decision-making can counterbalance top-down directives and lead to improvements in quality measurement. Third, training of family doctors by hospital specialists and integrating community and hospital services can assist in establishing patients’ trust in the quality of primary care. As China accelerates toward developing a national quality measurement program in primary care, more research is needed to consolidate what countries can learn from each other and how these lessons can be adapted to local contexts.

## Acknowledgements

 We would like to express our gratitude to all the interviewees who participated in this research and shared their thoughts and experiences with us. We would like to thank Academic Language Experts for their contribution to the manuscript.

## Ethical issues

 Ethical approval was obtained from the Institutional Ethics and Human Subjects Review Committee of Ben-Gurion University of the Negev (#32-2019). All participants provided informed consent.

## Competing interests

 Authors declare that they have no competing interests.

## Authors’ contributions

 AR conceived the study concept and design. All authors provided input into study design. AR and YP completed the data collection and qualitative analysis, through consultations and discussions with SH, MEE, ZT, JH, OM, and ND. All authors contributed to writing the original draft and approved the final manuscript.

## Supplementary files


Supplementary file 1. CFIR Interview Guide for In-depth Interviews With Stakeholders Involved in Providing, Managing and Regulating Diabetes Care in Shanghai (English Version).

Supplementary file 2. Interview Guide for Residents With Diabetes (English Version).

Supplementary file 3 contains Tables S1-S3 and Figures S1-S3.
