# Supplementary material for: Quality and Performance Measurement in Primary Diabetes Care: A Qualitative Study in Urban China
Source: Int J Health Policy Manag. 2022 Jun 7;11(12):3019–31. doi: 10.34172/ijhpm.2022.6372 (PMC10105207; doi:10.34172/ijhpm.2022.6372)
Supplement: Supplementary file 1 — CFIR Interview Guide for In-depth Interviews With Stakeholders Involved in Providing, Managing and Regulating Diabetes Care in Shanghai (English Version). [file ijhpm-11-3019-s001.pdf]

Article title: Quality and Performance Measurement in Primary Diabetes Care: A Qualitative Study in Urban China

Journal name: International Journal of Health Policy and Management (IJHPM)

Authors' information: Alon Rasooly[1]*, Yancen Pan[2], Zhenqing Tang[3], He Jiangjiang[3], Moriah E. Ellen[1], Orly Manor[4], Shanlian Hu[5], Nadav Davidovitch[1]

[1]School of Public Health, Ben-Gurion University of the Negev, Beer Sheva, Israel.

[2]Department of Epidemiology, Fielding School of Public Health, University of California, Los Angeles, CA, USA.

[3]Shanghai Health Development Research Center, Shanghai, China.

[4]Braun School of Public Health and Community Medicine, Hebrew University, Jerusalem, Israel.

[5]School of Public Health, Fudan University, Shanghai, China.

(*Corresponding author: Email: rasooly@post.bgu.ac.il)

Supplementary file 1. CFIR Interview Guide for In-depth Interviews With Stakeholders Involved in Providing, Managing and Regulating Diabetes Care in Shanghai (English Version).

Opening questions:

A. Can you please tell me about your work?
B. When treating diabetes, how do you see the differences between the work carried out by family doctors and by hospital specialists?
C. In your view, why would a patient prefer getting treated at a hospital or CHC?
D. What is the current situation of quality measurement for diabetes care in Shanghai?

Questions by CFIR dimensions and constructs

1. Process

1.1 Goal

- 1.1.1 What are the goals for improving diabetes care?
- 1.1.2 Why is quality measurement important in diabetes care?

1.2 Planning

- 1.2.1 Can you please describe the plan for implementing quality measurements in CHCs?
- 1.2.2 Who is involved in planning quality measures? What are their roles?
  - 1.2.2.1 In your view, who else should be involved?

1.3 External change agents

- 1.3.1 Will someone (or a team) outside your organization help you implement quality measurement? (question for CHC health provider or administrator)
- 1.3.2 What is your role in quality improvement initiatives for diabetes? (question for an external expert, such as hospital specialist)

1.4 Reflecting and evaluating

- 1.4.1 What kind of feedback do you use to evaluate current quality measurements?
- 1.4.2 Will you ask for feedback from staff? Or from residents that receive care?

2. Outer setting

2.1 Patient needs and resources

- 2.1.1 How well do you think that quality measurements meet the needs of patients with diabetes?

2.2 Cosmopolitanism

- 2.2.1 What professional networking do you engage in?
  - 2.2.1.1 Local, national, or international conferences? Training?
- 2.2.2 To what extent does your organization encourage you to network with colleagues outside your own setting?

2.3 Competition

- 2.3.1 Is there any competition or ranking associated with quality measures?
  - 2.3.1.1 If your unit ranked? If you rank other units?

2.3.2 What is your opinion on competition and ranking in the system?
   2.3.2.1 Would this bring more government funding to your center?
   2.3.2.2 What is the effect of competition on the care that patients receive?

3. Inner setting

3.1 Networks and communications
   3.1.1 Can you describe your working relationships with your colleagues?
   3.1.2 How is it for you to work as part of your team?

3.2 Culture
   3.2.1 How would you describe the culture of your organization? Your unit?
   3.2.2 How do you think your organization's culture will affect the implementation of quality measurements?

3.3 Relative priority
   3.3.1 In your work, what is the highest priority for you?
   3.3.2 What is of highest priority for your organization?
   3.3.3 How does the priority of implementing quality measures compare to other priorities in your organization? For your own work?

3.4 Organizational incentives and rewards
   3.4.1 What kinds of incentives are there for using quality measures in CHC?
   3.4.2 In case you are measured, to what extent good quality results may affect your promotion?

3.5 Feedback (relevant for CHC providers and administrators)
   3.5.1 Do you get any feedback reports about your work?
   3.5.2 Can you describe what these reports look like?
   3.5.3 How helpful are those reports? Can they be improved?
   3.5.4 How often do you get feedback on your work, and from who?

4. Intervention characteristics:

4.1 Trialability
   4.1.1 Do you think it would be possible to pilot quality indicators in a few CHCs?
   4.1.2 How should it be done?
   4.1.3 What costs will be incurred to implement quality measurements?
