# Supplementary material for: Quality and Performance Measurement in Primary Diabetes Care: A Qualitative Study in Urban China
Source: Int J Health Policy Manag. 2022 Jun 7;11(12):3019–31. doi: 10.34172/ijhpm.2022.6372 (PMC10105207; doi:10.34172/ijhpm.2022.6372)
Supplement: Supplementary file 2 — Interview Guide for Residents With Diabetes (English Version). [file ijhpm-11-3019-s002.pdf]

Article title: Quality and Performance Measurement in Primary Diabetes Care: A Qualitative Study in Urban China

Journal name: International Journal of Health Policy and Management (IJHPM)

Authors' information: Alon Rasooly[1]*, Yancen Pan[2], Zhenqing Tang[3], He Jiangjiang[3], Moriah E. Ellen[1], Orly Manor[4], Shanlian Hu[5], Nadav Davidovitch[1]

[1]School of Public Health, Ben-Gurion University of the Negev, Beer Sheva, Israel.

[2]Department of Epidemiology, Fielding School of Public Health, University of California, Los Angeles, CA, USA.

[3]Shanghai Health Development Research Center, Shanghai, China.

[4]Braun School of Public Health and Community Medicine, Hebrew University, Jerusalem, Israel.

[5]School of Public Health, Fudan University, Shanghai, China.

(*Corresponding author: Email: rasooly@post.bgu.ac.il)

Supplementary file 2. Interview Guide for Residents With Diabetes (English Version)

Living with Diabetes

1. Can you describe how you were diagnosed with diabetes?
   a. Who diagnosed your condition and where?
   b. How and when was it diagnosed?
   c. What did you do after being diagnosed?
2. How are you managing your diabetes?
   a. Have you made any changes in your diet and exercise activities?
   b. What drugs are you using and their frequency? By injection or per-os?
   c. Please describe consultations with your family doctor and specialists.

Receiving care at CHC and hospital

3. Can you describe your last visit to the CHC regarding diabetes?
   a. Why did you come?
   b. What were the doctor's recommendations?
4. Can you describe your last visit to a hospital regarding diabetes?
   a. Why did you come?
   b. What were the doctor's recommendations?
5. In what condition do you prefer CHC or hospital?
   a. Why would you prefer hospital care?
   b. What are the benefits of care in CHC?
6. Can you describe your interaction with your family doctor?
   a. How often do you meet with your family doctor? Are you in contact with him/her?
   b. What kind of health education (diet, exercise, smoking) have you received?
   c. What recommendations regarding testing?
   d. Were you able to follow these recommendations? (barriers: challenges to changing diet/smoking habits, tests/drugs too expensive)
   e. Do you use social media (WeChat) to interact with your family doctor or other patients?
7. Can you describe the referral process from the CHC to the hospital?
   a. Referral process from the hospital to the CHC?
   b. Have you had any trouble with the referral process? (waiting time, medical records)
   c. Repeated tests? Contradicting drug recommendation
8. Have you had a consultation with a specialist in the CHC? What are your thoughts about it?

Questions on policy

9. Why did you sign-up to receive care from a family doctor?
10. When living with diabetes, what kind of payments do you have?
    a. Who and for what services/products do you pay?
    b. Does the insurance reimburse you? What costs are still considered high?
    c. Are there any recommended tests or treatments that you did not take because of cost?
11. Were you asked to give feedback on the care that you receive?
    a. On the healthcare received by your family doctor? In hospital?
    b. In your view, why is patient feedback important?
12. How would you recommend improving the diabetes care that you receive?
13. What needs do you have that are not fully addressed?
