# Supplementary material for: Quality and Performance Measurement in Primary Diabetes Care: A Qualitative Study in Urban China
Source: Int J Health Policy Manag. 2022 Jun 7;11(12):3019–31. doi: 10.34172/ijhpm.2022.6372 (PMC10105207; doi:10.34172/ijhpm.2022.6372)

Article title: Quality and Performance Measurement in Primary Diabetes Care: A Qualitative Study in Urban China

Journal name: International Journal of Health Policy and Management (IJHPM)

Authors' information: Alon Rasooly[1]*, Yancen Pan[2], Zhenqing Tang[3], He Jiangjiang[3], Moriah E. Ellen[1], Orly Manor[4], Shanlian Hu[5], Nadav Davidovitch[1]

[1]School of Public Health, Ben-Gurion University of the Negev, Beer Sheva, Israel.

[2]Department of Epidemiology, Fielding School of Public Health, University of California, Los Angeles, CA, USA.

[3]Shanghai Health Development Research Center, Shanghai, China.

[4]Braun School of Public Health and Community Medicine, Hebrew University, Jerusalem, Israel.

[5]School of Public Health, Fudan University, Shanghai, China.

(*Corresponding author: Email: rasooly@post.bgu.ac.il)

Supplementary file 3.

Supplementary file 3

Table S1: Patients Interviewed in Shanghai, 2018-2019

Participants' organizational affiliations were coded to maintain confidentially.

Patient code	Setting	Age	Education attainment	Gender
**P01**	CHC1	68	Junior middle school	Female
**P02**		68	Junior middle school	Male
**P03**	CHC2	59	High school	Female
**P04**		77	Vocational education	Female
**P05**	CHC3	63	Junior college	Male
**P06**		65	Junior college	Male
**P07**	CHC4	80	Undergraduate	Male
**P08**		71	Undergraduate	Male
**P09**	Tertiary hospital 1	69	Junior high school	Male
**P10**		64	[unknown]	Male
**P11**	Tertiary hospital 2	65	Junior high school	Male
**P12**		77	Undergraduate	Male

Table S2: Providers and Policy Makers Interviewed in Shanghai, 2019

Code	Category	Organization	Gender
**S01**	Endocrinologist	Tertiary hospital 1	Female
**S02**		Tertiary hospital 2	Female
**FD01**	Family Doctor	CHC3	Female
**FD02**		CHC2	Female
**FD03**		CHC4	Female
**M01**	CHC manager	CHC3	Female
**M02**		CHC2	Male
**M03**		CHC4	Female
**M04**		CHC1	Male
**PM01**	Policy makers and senior managers	District health bureau	Male
**PM02**		District health insurance department	Female
**PM03**		Research center for diabetes policy	Female
**PM04**		Municipal Center for Disease Control	Male
**PM05**		Municipal health policy center	Male

Table S3. Facilitators and Barriers of Quality Measurement in Primary Diabetes Care in Shanghai Categorized According to CFIR Constructs

CFIR Construct	Facilitators	Barriers
**CFIR domain: process**		
Planning	Top-down planning allows for uniform implementation and evaluation of goal attainment on a national scale.	The exclusion of frontline clinicians from indicator planning may result in a mismatch between intended policies and actual context.
Reflecting & Evaluating	An online app allows directors and providers to reflect on their relative performance daily.	Lack of transparency – the app provides information to managers and providers but not patients.
**CFIR domain: inner setting**		
Goals	Quality indicators bridge national goals and family doctors' work.	Policies are directed by top-down goals, with insufficient bottom-up feedback.
Culture (organiza-tional)	A culture of team effort allows managers to increase cohesion and achieve goals.<br>Family doctors perceive their working environment as harmonious.	A rigid organizational culture leaves less room for the involvement of family doctors and patients in decisions on quality.
**CFIR domain: outer setting**		
External policy and incentives	Family doctors are financially rewarded for encouraging patients' quarterly visits and glycemic control. Monetary incentives and honors motivate to improve measured care.	Indicators are not adjusted for regional variation in incidence nor patients' social-demographic characteristics. Sometimes, incentives induce false reporting.
Patients' needs	CHCs improved at addressing patients' needs. Patients gradually establish a trusting and continuous relationship with their family doctors. The "signing policy" supports this change.	Distrust in family doctors' abilities still drives patients to tertiary hospital care. Current quality indicators are dissociated from how the patient experiences his/her health.
**CFIR domain: individual**		
Knowledge about intervention	Growing awareness regarding the advantages of CHC – encourages care by family doctors and enables quality improvement in CHCs.	Low awareness of CHC services still obstructs quality measurement and improvement in primary care.
Support of intervention	Family doctors support the quality evaluation system, perceiving it as a "scientific" and effective way for providing diabetes care.	Support may be limited due to the exclusion of frontline primary healthcare providers from decision processes.
**CFIR domain: intervention characteristics**		
Cost	Hospitalizations are expensive - better primary care for diabetes reduces medical costs for patients and insurers.	Quality indicators are associated with human resource and equipment costs - not funded by public insurance schemes.
Trialability	Evidence-informed indicators which were successfully implemented abroad can be first piloted in a subset of CHCs.	Local initiatives must be aligned with the national health policy discourse, limiting their flexibility.

Abbreviations: CHC - Community Healthcare Center, CFIR – Consolidated Framework for Implementation Research

Figures S1-S3: Thematic Networks - Produced with ATLAS.TI

Codes associated with "Organizational Incentives" & "External Policy and Incentives"

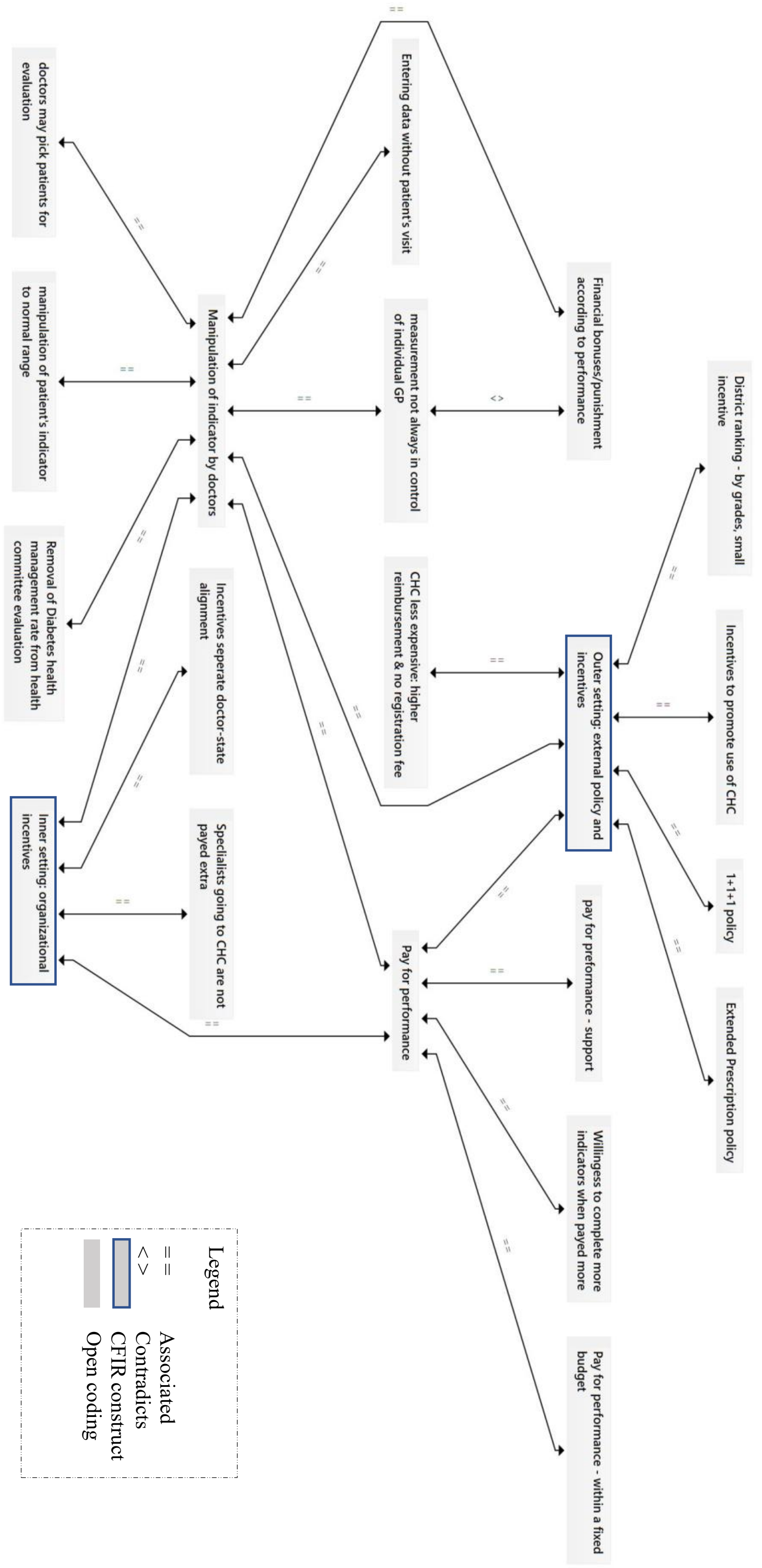

Codes associated with “Peer Pressure”

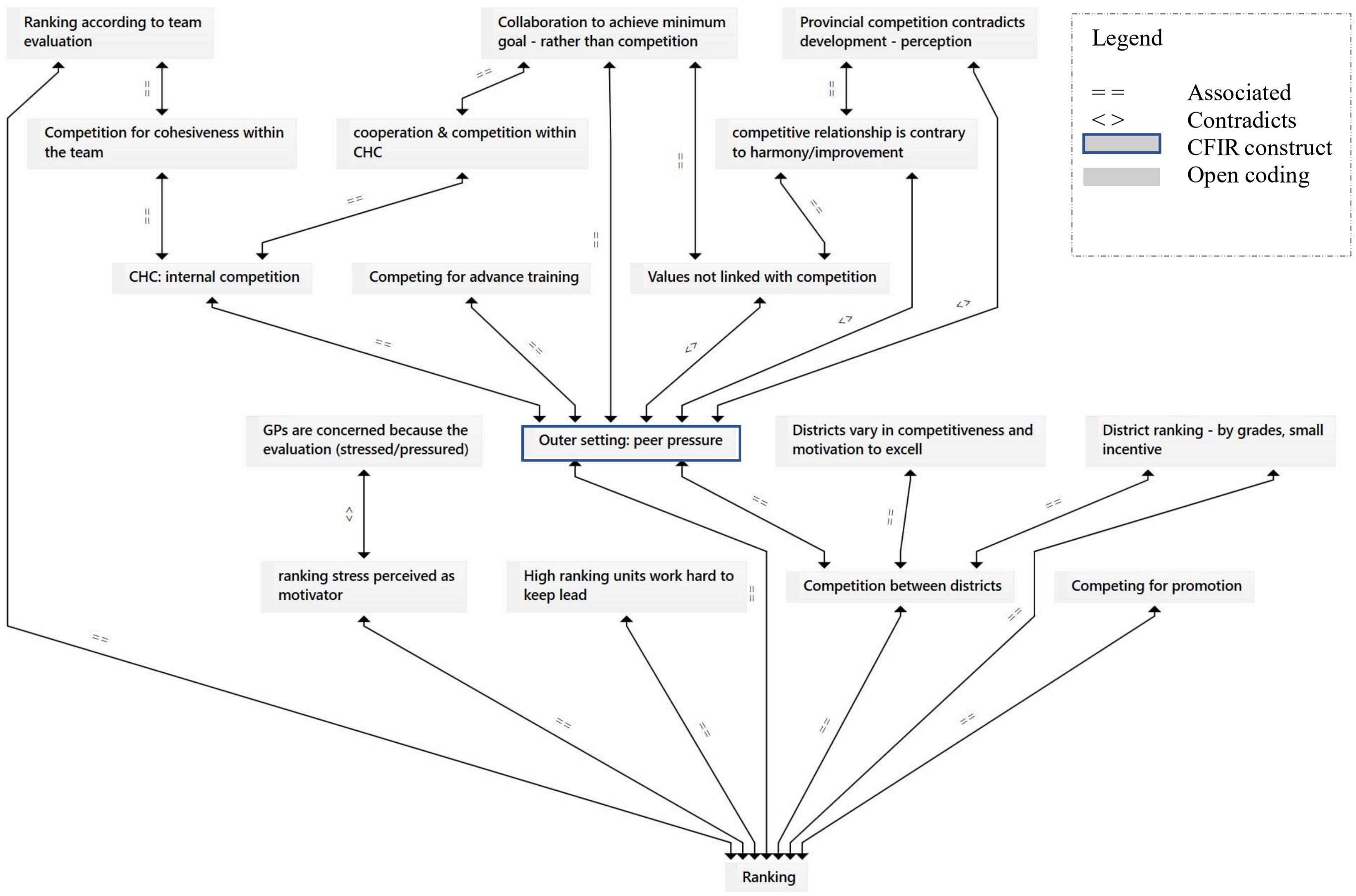

Codes associated with “Planning”

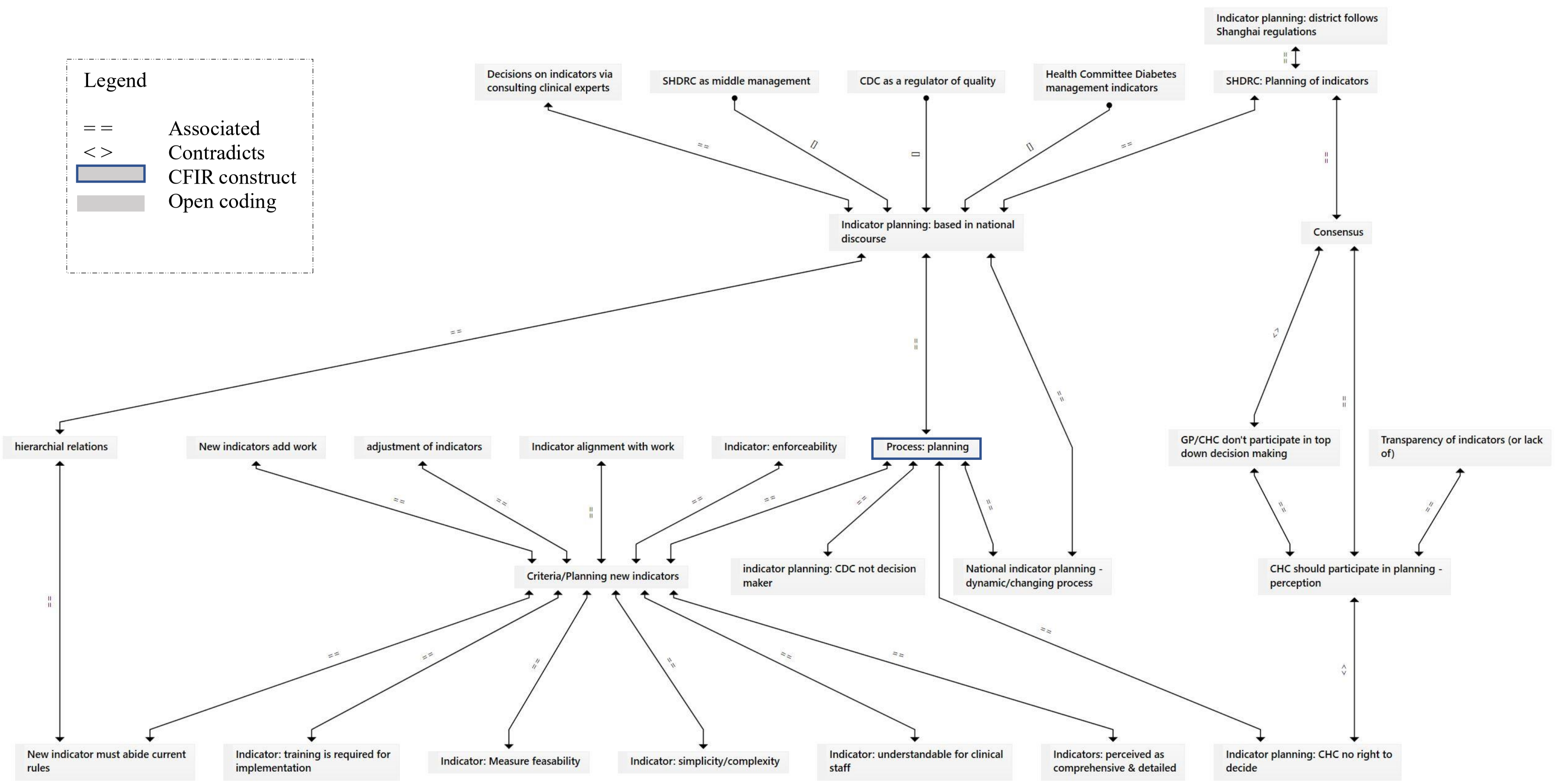

Supplement: Supplementary file 3 — contains Tables S1-S3 and Figures S1-S3. [file ijhpm-11-3019-s003.pdf]
